# Delamination and Evaluation of Multilayer PE/Al/PET Packaging Waste Separated Using a Hydrophobic Deep Eutectic Solvent

**DOI:** 10.3390/polym16192718

**Published:** 2024-09-25

**Authors:** Adamantini Loukodimou, Christopher Lovell, George Theodosopoulos, Kranthi Kumar Maniam, Shiladitya Paul

**Affiliations:** 1 Materials Innovation Centre, School of Engineering, University of Leicester, Leicester LE1 7RH, UK; 2 Materials Performance and Integrity Technology Group, TWI Technology and Training Centre, Middlesbrough TS2 1DJ, UK; 3 Materials Performance and Integrity Technology Group, TWI Ltd., Cambridge CB21 6AL, UK

**Keywords:** recycling, polymer, multilayers, flexible packaging, DES

## Abstract

This research concerns the development and implementation of ground-breaking strategies for improving the sorting, separation, and recycling of common flexible laminate packaging materials. Such packaging laminates incorporate different functional materials in order to achieve the desired mechanical performance and barrier properties. Common components include poly(ethylene) (PE), poly(propylene) (PP), and poly(ethylene terephthalate) (PET), as well as valuable barrier materials such as poly(vinyl alcohol) (PVOH) and aluminium (Al) foils. Although widely used for the protection and preservation of food produce, such packaging materials present significant challenges for established recycling infrastructure and, therefore, to our future ambitions for a circular economy. Experience from the field of ionic liquids (ILs) and deep eutectic solvents (DESs) has been leveraged to develop novel green solvent systems that delaminate multilayer packaging materials to facilitate the separation and recovery of high-purity commodity plastics and aluminium. This research focuses on the development of a hydrophobic DES and the application of a Design of Experiments (DoE) methodology to investigate the effects of process parameters on the delamination of PE/Al/PET laminate packaging films. Key variables including temperature, time, loading, flake size, and perforations were assessed at laboratory scale using a 1 L filter reactor vessel. The results demonstrate that efficient separation of PE, Al, and PET can be achieved with high yields for material and solvent recovery. Recovered plastic films were subsequently characterised via Fourier-transform infra-red (FTIR) spectroscopy, Differential Scanning Calorimetry (DSC) and Thermogravimetric Analysis (TGA) to qualify the quality of plastics for reuse.

## 1. Introduction

**Figure 1:**
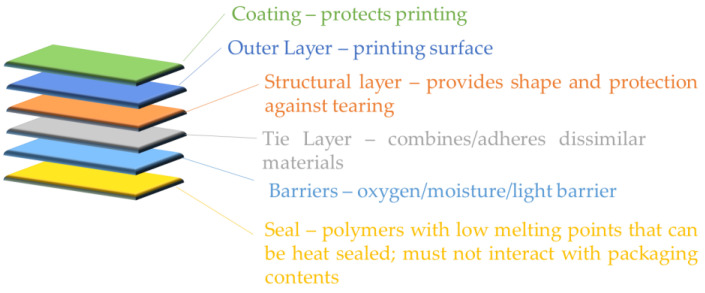
Multilayer packaging material inspired by [2].

Plastic laminate recycling is a challenging task due to the heterogeneous nature of plastic laminate’s intrinsic composition. The three main challenges of recycling plastic products are as follows: (a) the complexity and cost of the classification and separation of materials according to their composition and properties, especially, for the purpose of mechanical recycling; (b) the use of additives and coatings, such as reinforcing agents, antioxidants, plasticizers, dyes, inks, tie layers, metals, and flame retardants, which cannot be removed readily; and (c) contamination of plastic waste, including food packaging, which requires prior cleaning [1]. Typically, a laminate packaging film consists of different layers that are combined accordingly to broaden the functionality of the film (Figure 1) [2]. Laminate flexible packaging films are inexpensive, light, provide protection, can be easily moulded into different shapes and sizes, and are very durable [3]. Hence, they offer protection, extend the product lifetime, and also display individual product information [4]. However, increased numbers of layers and the inherent complexity of the structure usually lead to a reduction of material recyclability. Due to the complexity and incompatibility in the separation and recovery of the layers after use, landfilling appears to be the easiest and cheapest option [5]. A recent review by Li et al. shows the up-to-date progress in recycling this type of material [6]. However, the mismanagement of large production volumes that enter society leads to the conclusion that multilayer packaging films are a non-recyclable environmental pollutant. Worldwide, plastic waste management is a major problem—less than a fifth of plastic waste packaging is currently collected for recycling globally, with the remainder ending up in landfills, waterways, and oceans [7]. Plastics Europe reported that, in 2022, the total global output of plastic products was 400.3 Mt [8]. More than 90% of these plastic products are derived from fossil-based materials [9]. Towards the global direction of sustainable plastic production [10], microplastics reduction [11], and tackling climate change, efforts are gathered towards effective management and the recycling (mechanical/chemical) of plastics.

Various alternative chemical and physical approaches to recycling laminates have been developed, including cryo-comminution, chemical separation with hydrochloric and sodium hydroxide, organic solvents, hydrothermal processes, and switchable hydrophilicity solvents. Although some of these separation methods were partially efficient, they were abandoned due to high energy demands and concerns about non-ecofriendly properties [5]. Researchers have also extensively studied the delamination of laminate films [12], with several patented approaches, for example, one which uses organic solvents [13] to dissolve polymers and another with a solution comprising water, acetic acid, phosphoric acid, and sodium hydroxide [14]. Both of these suffer from a relatively slow delamination. More recently, another patent disclosed the use of perforations to accelerate the delamination process for multilayer laminates using a caustic 10% sodium hydroxide solution, including both cationic and anionic surfactants [15]. Researchers have also published a study demonstrating efficient delamination of perforated packaging films comprising aluminium barrier layers, either as foils or metalised films, in less than 15 min at temperatures of 50, 65, and 80 °C [16]. Carboxylic acids are also well understood to efficiently delaminate multilayer packaging via diffusion of dimers through the films to the adhesive tie layers, which swell and dissolve, facilitating the separation of the component films [17,18,19]. Üdülger et al. presented a detailed study on the mechanism of delamination with low-molar-mass carboxylic acids, including formic acid, acetic acid, hexanoic, and decanoic acid [4].

Towards the development of a green chemistry approach, deep eutectic solvents offer a promising solution, paving the way to packaging waste delamination, recovery, and recycling of the separated components.

Deep eutectic solvents (DES) are a group of mixtures of Lewis or Brønsted acids and bases that form stable solutions at room temperature. DES first appeared in the literature in 2001 [20] with choline chloride and urea/carboxylic acids [20,21] attracting great interest. The release of the first major review concerning developments in the field, including physical and chemical properties as well as applications, was presented in 2014 by Abbott et al. [22]. In the last decade, a great deal of interest has been gathered on the topic, with numerous reviews being released, for example, on separation applications [23], hydrophobic deep eutectic solvents (HDES) in microextraction techniques [24], the background of DES [25], and physicochemical properties of deep eutectic solvents [26]. In summary, DES systems have been extensively studied for a wide variety of processes, such as electrolytic, immersion, electroless deposition, and electropolishing. Further, they have also been shown to be used as specific extractants (e.g., for the purification of biodiesel) and suitable solvents for a number of other processes, such as liquid–liquid extraction and gas capture and synthesis [27].

Since 2015, intense interest has been shown in hydrophobic deep eutectic solvents (HDES), which can be explained by their favourable properties, such as density, viscosity, acidity or basicity, polarity, low volatility, low toxicity, and good extraction capabilities for various target analytes. Through the appropriate selection of hydrogen bond donors (HBD) and hydrogen bond acceptors (HBA), new HDES could be formed. These systems are considered as a new generation of DES [25] and represent promising alternatives to the traditional organic solvents. Moreover, the possibility of using non-toxic ingredients for their preparation lists HDES as an important candidate for future eco-friendly solvents [24]. During the interaction of HBA and HBD components, extensive hydrogen bond interactions are formed and decrease the melting point of the mixture [28]. Due to this bond formation ability between HBD and HBA, most DESs are generally hydrophilic and dissolve rather easily in water. The hydrophobicity of DES is strongly affected by the structure of individual components HBD and HBA [24].

Recycling of PE/Al/PET waste films requires the purification and separation of the constituent materials. Poly(ethylene) (PE) and poly(ethylene terephthalate) (PET) are commonly used polymers in food packaging, maintaining their high mechanical strength up to and above 100 °C. PE is difficult to recycle by chemical processes because its C–C covalent bonds require high temperatures and high-performance catalysts for their decomposition. On the other hand, PET, a polymer with an ester-based polymer chain, requires hydrolysis for its decomposition. Researchers claim that sorting mixtures of plastic waste is challenging with low sorting efficiency. However, the developed solvent system in this research gives a promising solution for the separation of PE/Al/PET film at temperatures lower than 100 °C with low energy demands [1].

In this paper, we present experiments on a PE/Al/PET laminate packaging waste using an HDES solvent system, which shows promising results and great potential to be applied to the delamination of multilayer packaging waste. By testing variables such as time, agitation rate, size, and temperature, the optimization of the process and the analysis provide new insights into the field of recycling.

## 2. Materials and Methods

### 2.1. Solvent

Recent developments concerning a wide range of HDESs were discussed by Zainal-Abidin et al. [29], with a particular focus on the chemistry of different formulations published in the literature. To the best of our knowledge, the solvent system employed in this research has not previously been investigated.

The two chemical components of the DES formulation, an H-bond donor (≥99% purity) and an H-bond acceptor (≥99% purity), were purchased from Merck Life Science UK Ltd. and used without further purification. The hydrophobic character of the solvent was imparted by the selection of the H-bond acceptor which exhibited poor miscibility with water. The resulting combination of H-bond donor and acceptor chemicals in a 1:1 molar ratio for DES formulation was also found to be immiscible with water.

Three 1 kg batches of the DES formulation were prepared for the series of experiments. After the molar quantities of chemicals had been weighed out into a 1 L glass bottle, the mixture was heated to 70 °C and agitated using a VELP AREX 6 Digital Hot-plate Stirrer until the formation of a homogeneous transparent liquid.

### 2.2. Packaging Laminate

**Figure 2:**
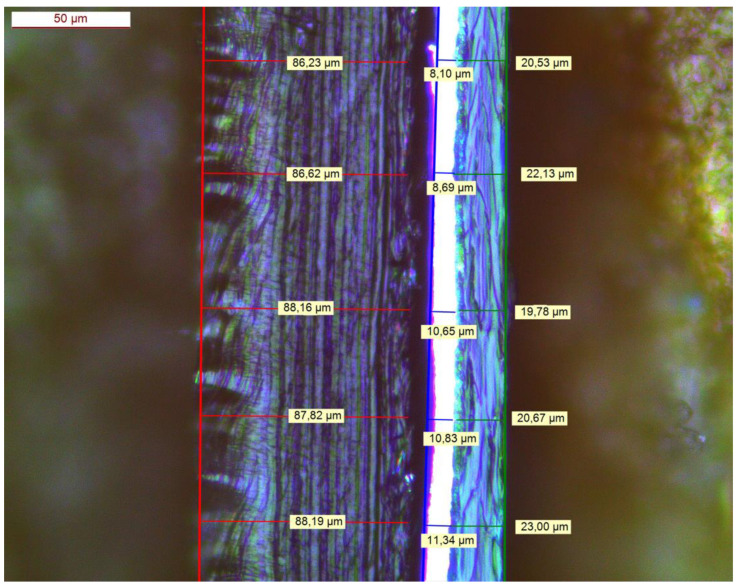
Cross-section of the PE/Al/PET laminate (PE—red; Al—blue; PET—green).

A quantity of commercial PE/Al/PET packaging film obtained from a primary waste stream was supplied by Plastigram Industries A.S (Tovární, Czechia). As shown in Figure 2, the three-layer construction comprised a PE film of thickness 87.4 ± 0.9 μm, an alumimium foil of thickness 9.9 ± 1.4 μm, and a reverse-printed PET film of thickness 21.2 ± 1.3 μm. The adhesive layer between the PE and Al layers was determined to have a thickness of 9.8 ± 1.1 μm.

To evaluate the effect of increased accessibility of the solvent to the ink and adhesive layers, a hand perforation tool (J. Clark & Co., Ltd., Shipley, UK) was used to pierce a proportion of the PE/Al/PET material. The tool comprised 2904 pins of Ø = 0.88 mm arranged in a diamond lattice with a density of (approx.) 7 pins cm^−2^.

For the study, both perforated and non-perforated samples of the packaging film were cut into square pieces of (approx.) 10 mm, 30 mm, and 50 mm dimensions.

### 2.3. Delamination

All delamination trials were performed in a 1 L jacketed filter reactor vessel (R.B. Radley & Co., Ltd., Saffron Walden, UK) equipped with a digital torque overhead stirrer (Heidolph Instruments GmbH, Schwabach, Germany), a heater–chiller circulator for temperature control (Huber UK Temperature Control Ltd., Ripley, UK), and a chemical vacuum pump (Fisher Scientific UK Ltd., Loughborough, UK) to drain the solvent through the filter plate into a receiving flask. The filter plate was fitted with a P100 sintered glass disk (pore size 40–100 μm) with a 10 μm pore size PET filter membrane secured atop.

AVA Lab Control software V2.0.3 (R.B. Radley & Co., Ltd.) was employed to set up test programmes and to set the experimental parameters of solvent temperature, stirrer speed, and step duration for each test. This ensured reproducibility of the process and control over the experimental conditions in each trial. A period of equilibration at the target temperature, typically ~1 h, was allowed prior to commencing each delamination trial, which started upon the addition of PE/Al/PET material to the vessel. Delamination experiments were run at set temperatures and for set periods of time according to a Design of Experiments matrix of test conditions (Section 2.4). At the end of the delamination period, the vessel and contents were cooled over a period of 15 min before drawing the solvent through the filter base into a receiving flask. Following the solvent removal, the films retained in the vessel underwent four rinse cycles prior to being extracted.

The mix of delaminated PE, aluminium, and PET films was transferred to a container and stored in a fume cupboard under extraction at ambient temperature until the materials had dried, typically ~72 h. Characterisation of the PE and PET was then performed using Fourier-Transform Infra-red (FTIR) Spectroscopy, Differential Scanning Calorimetry (DSC), and Thermogravimetric Analysis (TGA).

Finally, after each trial, the quantity of solvent recovered from the vessel was measured to determine the extent to which the solvent was retained by the mass of delaminated materials. The solvent was then replenished up to 1 kg and re-used for further delamination trials performed at the same experimental temperature.

### 2.4. Design of Experiments

A Design of Experiments (DoE) approach was taken to evaluate the effects of temperature, time, size of the packaging pieces, loading, and film perforations on the extent of delamination using Design Expert V13 software (Stat-Ease, Inc., Minneapolis, MN, USA) to generate a suitable test matrix. Within the constraints of the parameter ranges set out in Table 1, a Definitive Screening Design (DSD) defined 14 different experimental conditions to assess via delamination trials.

DSDs are Resolution IV experimental designs, which means that the main effects (A, B, C, etc.) are not aliased with any 2-way interaction terms (AB, AC, BC, etc.). Therefore, they are ideal to use when main effects dominate the system response, and 2-way interactions do not have a significant influence on the experiments. Although 2-way interactions can be identified via DSDs, they may be confounded with other 2-way interactions and quadratic terms. In contrast to conventional 2-level Factorial Designs with Resolution IV, DSDs incorporate design points at three levels, that is, low-, high-, and mid-point values for each factor. As a result, under appropriate circumstances, significant quadratic terms (A^2^, B^2^, C^2^, etc.) may also be evaluated [30,31].

The individual trial conditions are shown in Table 2. Each experiment followed the procedure described above (Section 2.3), with the temperature and time of the delamination step adjusted accordingly. As mentioned, three batches of solvent were prepared in this study, one for each set of experiments performed at 60 °C, 70 °C, and 80 °C.

The response measured to quantify the extent of delamination was a count out of 100 non-delaminated, partially delaminated, and fully delaminated aluminium pieces taken from a sample of mixed film pieces. In each instance, the count was repeated with a second sample of 100 aluminium-containing pieces and an average was taken of the results. A logistic regression model was then applied to analyse the count data and probability of delamination.

### 2.5. Fourier-Transform Infrared Spectroscopy (FT-IR)

Fourier-Transform Infra-red (FTIR) Spectroscopy is an analytical technique for evaluating the chemical composition of materials. Typically, polymers will have their own characteristic infra-red spectrum, which reflects the combination of chemical groups in the material, such as C-H, C = O, C-O-C, and O-H. Therefore, the FTIR spectrum can be used to confirm a material or identify the presence of other materials. In the case of packaging materials, adhesives and inks also used in the assembly of the laminate structures will contribute to FTIR spectra if they remain on the surface of the PE, PET, and aluminium foils.

A 4300 Handheld FTIR Spectrometer (Agilent) equipped with a diamond-ATR and a resolution of 4 cm^−1^ was used to analyse PE and PET films. The number of scans for each acquisition was 16 and the scan range was 4000–650 cm^−1^.

### 2.6. Differential Scanning Calorimetry (DSC)

Differential Scanning Calorimetry (DSC) is a thermal analysis technique used to detect and quantify changes in the morphology of materials when heated and cooled, for example, to measure glass-transition temperatures and melting and crystallisation transitions in polymers. Experiments on the recovered plastics were performed using a DSC 214 Polyma (Netzsch Thermal Instruments UK Ltd., Wolverhampton, UK) under an inert atmosphere of N_2_ purge gas (40 mL min^−1^). Calibrations of the instrument temperature and heat flow were made using high-purity standards of indium (99.999%), tin (99.999%), bismuth (99.999%), zinc (99.999%), and caesium chloride (99.999%). Small quantities of each plastic film were placed in aluminium pans with masses in the range of 9–13 mg for PE and 5–11 mg for PET. Tests were performed via a temperature ramp from 25 to 300 °C at 10 °C min^−1^.

### 2.7. Thermogravimetric Analysis (TGA)

Thermogravimetric Analysis (TGA) is a thermal analysis technique used to determine the thermal stability of polymers. Experiments on the recovered plastics were performed using a TG 209 F1 Libra (Netzsch) under an inert atmosphere of N_2_ purge gas (40 mL min^−1^). Calibrations of the instrument temperature were made using high-purity standards of indium (99.999%), tin (99.999%), bismuth (99.999%), zinc (99.999%), and aluminium (99.999%). Small quantities of each plastic film were placed in aluminium pans with masses in the range of 8–13 mg for PE and 9–13 mg for PET. Tests were performed via a temperature ramp from 25 to 700 °C at 10 °C min^−1^.

## 3. Results and Discussion

### 3.1. Effect of Temperature, Time, Loading, Flake Size, and Perforations

Sampling of 100 aluminium-containing pieces from the mix of delaminated films yielded only three outcomes, that is, PE/Al/PET (non-delaminated), Al/PET (partially delaminated), and Al (fully delaminated) films. Pieces of PE/Al were not observed, as the PE film always separated on a shorter timeframe than the printed PET film. Using the delamination count for pieces containing aluminium, it can be inferred that the proportion of fully delaminated PE is equivalent to the sum of Al/PET and Al counts (or 100—PE/Al/PET counts) and that the proportion of fully delaminated PET is equal to the Al count. It was observed from the repeat assessments that the counts deviated by between 4 and 12 across PE/Al/PET, Al/PET, and Al pieces over all experiments. The variation produced by the sampling method was not significant compared to the full range of results produced by the different conditions. The full table of the delamination trial results is presented in Table 2.

Using Design Expert V13, linear models based on the Logit function were fit to the datasets (Equation (1)) as follows:

F(p_i_) = ∑_i_ ln ( p_i_/(1 − p_i_)) = ∑_i_ β_A_ A_i_ + β_B_ ∙B_i_ + β_C_ C_i_ + β_D_ D_i_ + β_E_ E_i_ + ε_i_ (1)

where the probability of delamination, p_i_, for a given set of experimental conditions, i, was defined as equal to the fraction of the delamination counts of 100. The software employed logistic regression (maximum likelihood estimation) to evaluate the significance of the model coefficients, β_f_, for each factor, f.

It may be noted that Equation (1) describes a linear model based on the main effects without additional 2-way interactions or quadratic terms that could otherwise be incorporated into the analysis. This model was found to provide an adequate description of the experimental results, and, therefore, it was decided not to expand the equation to include higher-order terms, which could result in overfitting of the data.

A summary of which factors proved to be significant within the experimental design space for the delamination PE and Al (or PET) is given in Table 3 (nb. factors with *p*-values below the threshold, α = 0.05, are given statistical significance).

**Figure 3:**
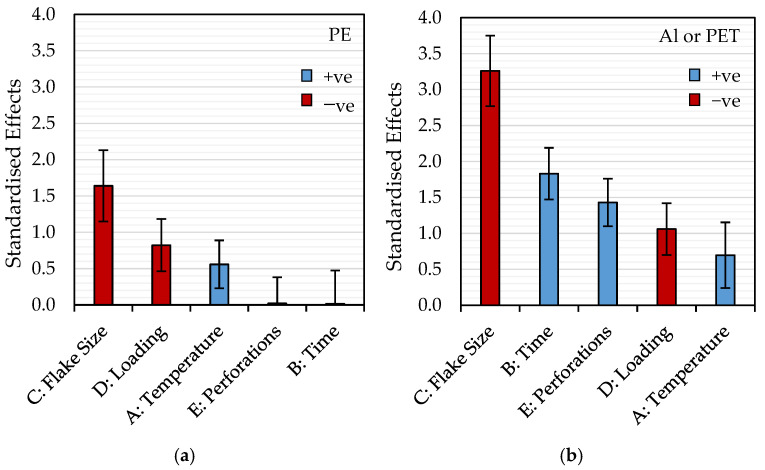
Pareto charts comparing the magnitudes of the standardized effects of each factor in the probability models for delamination of (**a**) PE and (**b**) Al or PET.

In Figure 3, Pareto charts show the relative influence (ranking) of each factor on the probability of delamination for PE and PET via the absolute values of standardized effects determined from the logistic regression. The standardized effects correspond to the coefficients of the model (Equation (1)) fit to the data sets in a coded design space where each factor is renormalized so that the parameter values range from −1 to +1. Factors with positive coefficients increase the likelihood, whilst conversely, those with negative coefficients reduce the likelihood of delamination under a given set of conditions. As can be seen, flake size and loading have negative coefficients in both PE and PET models and, therefore, reduce the probability of delamination, whilst temperature, time, and perforations yield positive coefficients promoting delamination. Interestingly, it can be seen from the analysis that flake size has the most significant influence on the success of delamination in these experiments. As noted in Table 3, the very low values of the standardized effects for perforations and time in the model for PE delamination (Figure 3a) highlight that these factors do not have a statistically significant effect on the outcome of the process within the range of experimental conditions investigated.

**Figure 4:**
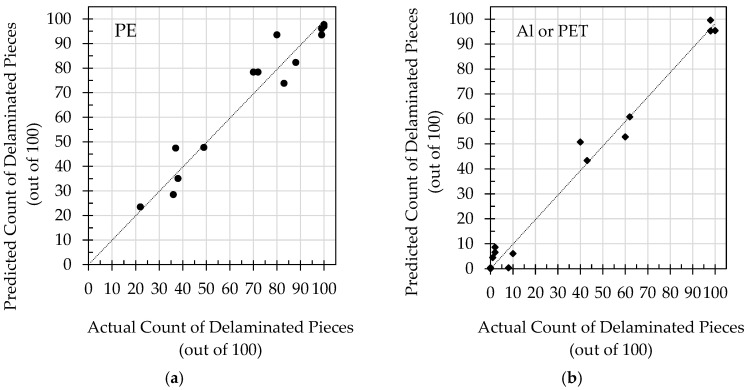
Plots comparing the predictions of the probability models with actual observations for the delamination of (**a**) PE and (**b**) Al or PET.

Figure 4a,b demonstrate strong correlations between the model predictions for the delamination of PE and Al (or PET) and actual counts of delaminated pieces obtained from the experiments.

The results and interpretation of models are further discussed in the following sections.

### 3.2. Analysis of Delamination Models

#### 3.2.1. Model for PE Delamination

Interestingly, neither time nor perforations were found to be significant for the delamination of PE from PE/Al/PET. A model based on temperature, flake size, and loading adequately predicted the probability of delamination. That implies the absence of any significant packaging interactions, which depend on the load and flake size of the film pieces. PE could be expected to fully delaminate in the temperature range from 60 to 80 °C within a timeframe of 15 min or less. For the 10 mm flake size, delamination of PE was essentially complete after 15 min, whilst for larger size pieces, and especially at higher loadings, physical hindrance likely dominated and inhibited clean separation of the layers. Given that perforations were not deemed to be significant, it supports the conclusion that the delamination of PE from the aluminium was driven by diffusion of the solvent through the film to swell and weaken the adhesive [4], as opposed to ingress from the film edges.

**Figure 5:**
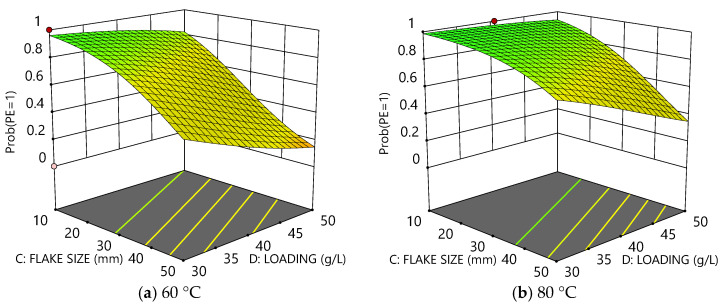
Plots comparing the probability of delamination for PE as a function of flake size and loading at (**a**) 60 °C and (**b**) 80 °C.

Figure 5a,b compare the probability surfaces for PE delamination at 60 °C and 80 °C, respectively. The plots illustrate how an increase in flake size and/or loading reduces the probability of delamination, but also how higher temperature can improve the overall probability of delamination.

#### 3.2.2. Model for Al or PET Delamination

**Figure 6:**
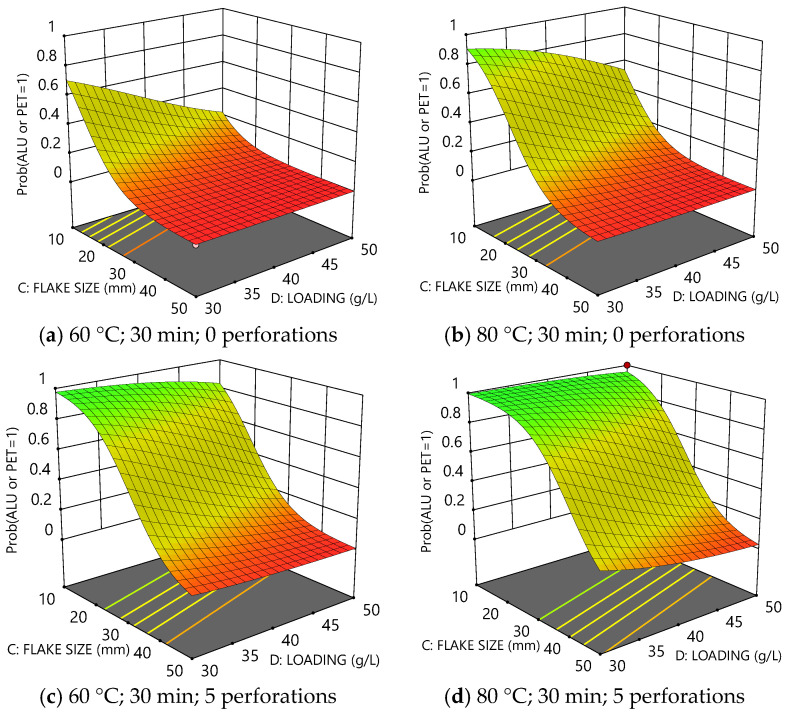
Plots comparing the probability of delamination for Al or PET as a function of flake size and loading after 30 min: (**a**) at 60 °C without perforations, (**b**) at 80 °C without perforations, (**c**) at 60 °C with perforations, and (**d**) at 80 °C with perforations.

The probability model for the delamination of PET from Al incorporates all five experimental factors. The four surface plots in Figure 6 collectively show how the probability varies as a function of flake size and loading both at 60 °C and 80 °C and also provide comparisons between as-received and perforated films. Again, increased size and loading of the film pieces reduced the probability of delamination for a given temperature and time. Increasing temperature accordingly increased the probability of delamination (e.g., compare Figure 6a,b). However, perforations can be seen to have an even more substantial effect than temperature (e.g., compare Figure 6b–d), dramatically increasing the probability of delamination.

It is evident that perforations greatly assisted the separation of PET and Al and had a clear benefit concerning the delamination mechanism [16]. Unlike the PE layer, the PET layer was reverse-printed with inks, which must be largely removed by the solvent to achieve separation of PET from the aluminium. Perforations allowed the solvent multiple points of access to the ink across the surface of each piece. In comparison, for unperforated films, the solvent could only ingress from the film edges, which yielded inherently slower rates of ink removal and delamination.

#### 3.2.3. Optimisation of PE/Al/PET Delamination

Optimisation of the delamination conditions can be achieved by defining a suitable set of desirable criteria. One optimisation scenario would be to maximise the probability of full delamination of the packaging film whilst minimising the temperature and time of the process. It would also be beneficial to maximise the flake size and loading of materials in the vessel.

**Figure 7:**
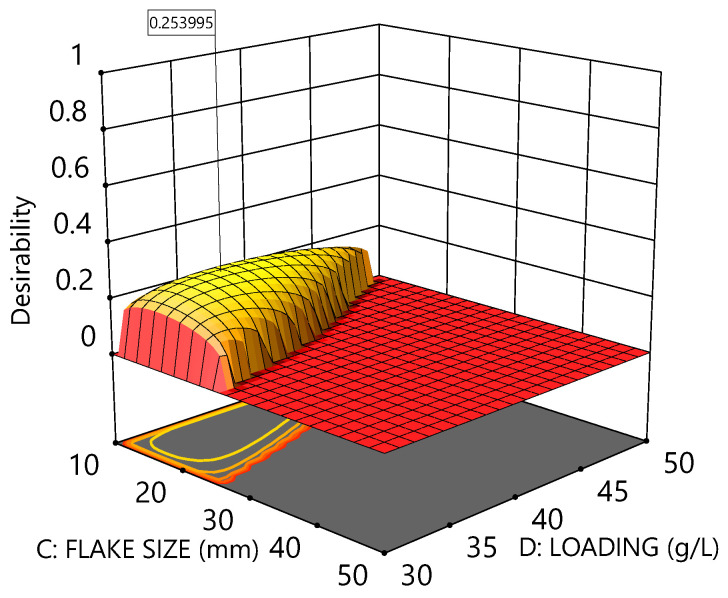
Desirability plot according to the optimisation criteria defined in Table 4. Optimal conditions correspond to Temperature = 72 °C, Time = 33 min, Flake size = 14 mm, Loading = 36 g L^−1^, and Perforations = 7 pins cm^−2^.

As an example, a list of such desirability criteria is given in Table 4 with the optimal conditions and predicted outcomes (based on those criteria) recorded in the final column. The corresponding desirability plot is shown in Figure 7.

The desirability plot above demonstrates that there is a small amount of leeway to increase both the flake size and loading whilst retaining a high probability of full delamination, *p* > 0.9, under the constraint that temperature and delamination time remain at 73 °C and 24 min, respectively.

### 3.3. Recovery of Solvents and Materials

The recovery of solvents and materials represents another important measure of the quality of the process. On average, 870 ± 20 g of solvent formulation was recovered after each experiment (87 ± 2%), with the remaining solvent retained by the mass of the film. The dried masses of delaminated films compared well with the initial loadings and demonstrated a high recovery yield. Where the measurement of the recovered mass of film was found to be a few percent greater than the initial loading (Table 2), it is evident that some residual solvent remained and, therefore, drying conditions could be further improved. A summary of mass measurements is given in Table 5.

### 3.4. FTIR Spectroscopy

**Figure 8:**
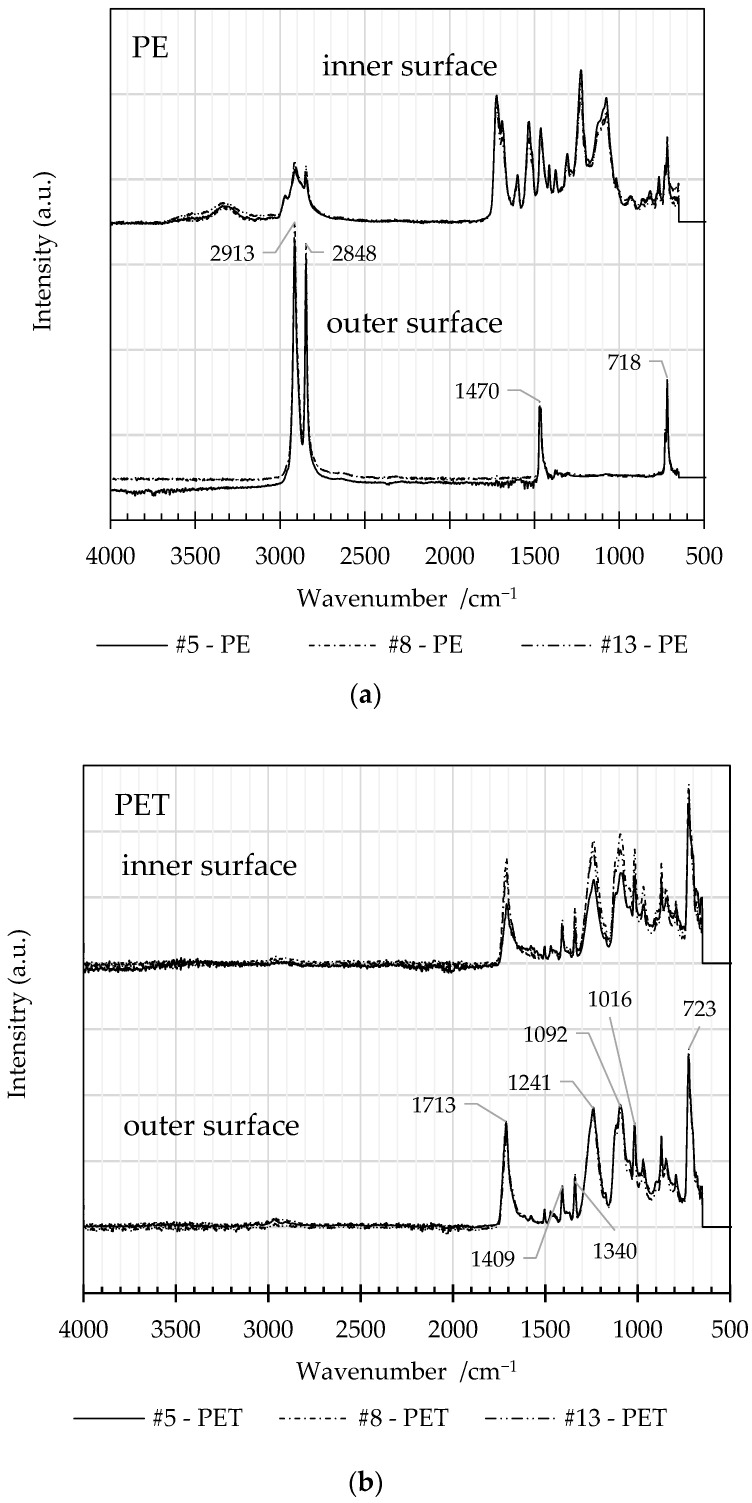
Comparison of the FTIR spectra obtained from the inner and outer surfaces of (**a**) PE and (**b**) PET films from delamination trials #5, #8, and #13.

Figure 8 presents FTIR spectra of the inner and outer surfaces of delaminated PE and PET from experiments #5, #8, and #13, performed at 60 °C, 70 °C, and 80 °C, respectively.

The inner surfaces of the PE films retained a component of the adhesive layer as evidenced by the contrast between spectra from inner and outer surfaces. The FTIR spectra of the outer surface are typical of PE with four bands at 2913, 2848, 1470, and 718 cm^−1^ due to C-H stretches and CH_2_ bending and rocking modes [32]. No carbonyl peak is observed at ~1720 cm^−1^, which would otherwise be indicative of oxidation.

The spectra from the inner and outer surfaces of the PET film are consistent with the standard material spectrum, as nearly all traces of the ink were removed from the inner surface by the solvent during the delamination step. The spectral bands at 1712, 1241, and 1092 cm^−1^ are characteristic of C=O stretch, C-C-O asymmetric stretch (aromatic), and C-O-C stretch modes associated with the ester group [33].

An overview of the assignment of the spectral absorption bands is given in Table 6. Importantly, FTIR spectra do not reveal any significant contribution from residual solvent within the films which must be present only at levels below the detection threshold.

### 3.5. Thermal Analysis

**Figure 9:**
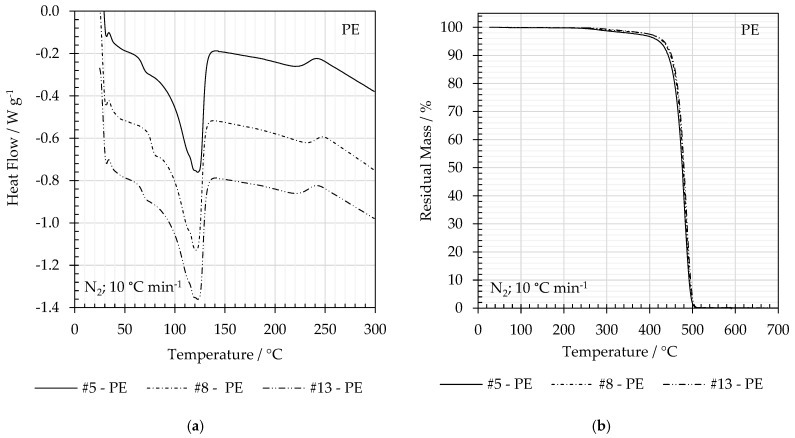

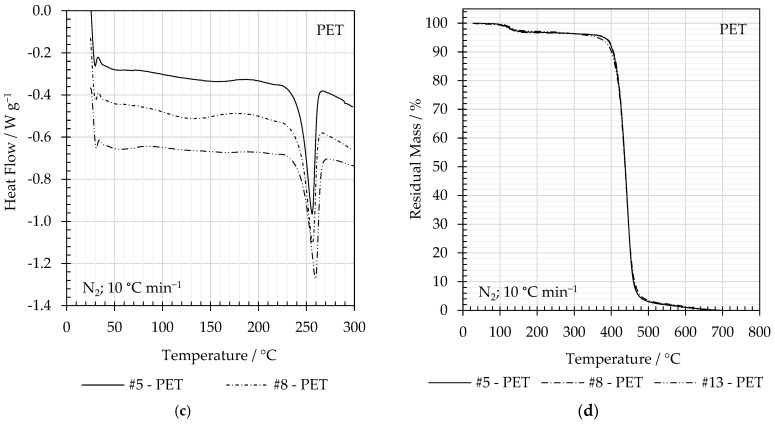
Comparison of the (**a**) DSC for PE, (**b**) TGA traces for PE, (**c**) DSC for PET, and (**d**) TGA traces for PET films from experiments #5, #8, and #13 and 60 °C, 70 °C, and 80 °C.

Figure 9 shows the DSC and TGA traces for PE and PET films from experiments #5, #8, and #13, and 60 °C, 70 °C, and 80 °C, respectively. The peak melting temperatures (T_m_) and enthalpies of fusion (ΔH_m_) from DSC as well as the onset thermal degradation temperatures (T_d_) are given in Table 7.

Both the DSC and TGA traces suggest a few percent loss of volatile material. In Figure 9a,b, there are step changes in the DSC heat flow and TGA mass for PE in the temperature range from 200 to 300 °C. The mass change at 350 °C is ~2–3% *w*/*w* before the onset of thermal degradation at ~460 °C. Figure 9d clearly identifies a mass loss of ~3–4% *w*/*w* for PET above 100 °C, which may be a combination of residual solvent and absorbed moisture. These coincide with broad features in the DCS trace between ~80 and 180 °C. The onset of thermal degradation for PET is ~416 °C.

## 4. Conclusions

In the present work, a novel hydrophobic ‘green’ solvent system has been developed for the separation and recovery of commodity materials such as PE, PET, and aluminium from flexible laminate packaging waste.

A Design of Experiments study for the delamination of PE/Al/PET packaging established that temperature, flake size, and loading were significant factors when modelling the probability of PE delamination within a 15- to 45-minute timeframe at 60 °C, 70 °C, and 80 °C. Delamination was more efficient when using smaller pieces (10 mm) at lower loading (30 g/L) compared to either larger pieces (up to 50 mm) or higher loadings (up to 50 g/L) (or both). The introduction of perforations did not have a significant impact on the rate of delamination for PE, which is evidently dependent on the permeation of solvent through the film [4]. In contrast, the delamination of PET from aluminium was strongly influenced by perforations, which significantly increased the probability of delamination for a given set of conditions. This is likely due to increased access of the solvent to the printed ink on the inner face of the PET bonded to the aluminium [16]. Overall, the probability of PET delamination increased with temperature and time but, again, decreased with increased flake size and/or loading. The combined effects of flake size and loading likely reflect inhibition of delamination due to the increased packing of film pieces in the 1 L vessel. These findings could feed into a scale-up procedure in future applications.

The FTIR spectra were overall consistent with the polymer reference spectra. It is noteworthy that any residual solvent was present at levels below the detection threshold for FTIR. A deviation from the standard spectra was detected on the inner surface of PE films due to the presence of adhesive residue. Spectra obtained from the inner and outer surfaces of PET films were consistent with library standards.

Thermal analysis of the recovered PE and PET films using DSC and TGA demonstrates that the morphology of the films is unaffected by the solvent-based process and that the films retain good thermal stability. A small amount of ~2–3% *w*/*w* of residual solvent and moisture was retained within the materials.

In conclusion, the characterisation of films by FTIR, DSC, and TGA demonstrated that the chemistry, semi-crystalline morphology, and thermal stability of the polymers were not significantly altered by the exposure to the solvent; all parameter values were typical of PE and PET. Whilst the films retained a small amount of residual solvent, this should only have a temporary physical influence on the polymers due to the permeation of the solvent through the amorphous regions. It is highly likely that in subsequent re-processing steps via the polymer melts, any remaining solvent would be volatilised, returning the material to its original condition. However, it is recognised that further scale-up and evaluation of reprocessed materials is required to assess recycled material properties and performance in detail and validate the overall quality of the products.

It is expected that with further development and scale-up, the technology demonstrated in this research can contribute to the recovery of high-purity polymers suitable for high-value end applications and reintegration into a circular economy.

## Figures and Tables

**Table 1:** Process parameters evaluated in the delamination study.

Parameters	Units	Type	Low	Centre	High
Temperature	°C	Numeric	60	70	80
Flakes Size	mm	Numeric	10	30	50
Loading	g	Numeric	30	40	50
Perforation	pins cm^−1^	Categoric	0	n/a	7
Time	min	Numeric	15	30	45

**Table 2:** Delamination trial conditions and results.

	Factors					Delamination Count			
#	A: Temperature (°C)	B: Time (min)	C: Flake Size (mm)	D: Loading (g)	E: Perforations (pins cm^−2^)	PE/Al/PET	Al/PET	Al or PET	PE
1	60	30	50	30	0	51	49	0	49
2	60	15	10	50	0	12	87	1	88
3	60	15	50	40	5	65	27	8	35
4	60	45	30	50	0	53	35	2	37
5	60	45	10	30	5	1	1	98	99
6	70	30	30	40	0	28	62	10	72
7	70	45	50	50	5	78	20	2	22
8	70	30	30	40	5	30	10	60	70
9	70	15	10	30	0	0	57	43	100
10	80	45	10	40	0	0	0	100	100
11	80	15	50	50	0	62	38	0	38
12	80	45	50	30	5	17	21	62	83
13	80	15	30	30	5	20	40	40	80
14	80	30	10	50	5	1	1	98	99

**Table 3:** Summary of significant factors in the linear models for the delamination of PE and Al (or PET) from PE/Al/PET.

	Factor *p*-Values					
Material	Model	A: Temperature (°C)	B: Time (min)	C: Flake Size (mm)	D: Loading (g)	E: Perforations (pins cm^−2^)
PE	<0.0001	<0.0001	0.855	<0.0001	<0.0001	0.782
Al or PET	<0.0001	0.0036	<0.0001	<0.0001	<0.0001	<0.0001

**Table 4:** An example of desirability criteria for delamination of PE/Al/PET and optimal conditions for the 1 L reactor vessel.

Factors and Responses	Criteria	Accepted Range		Optimal
		Min	Max	
A: Temperature (°C)	Minimise	60	80	72
B: Time (min)	Minimise	15	45	33
C: Flake size (mm)	Maximise	10	50	14
D: Loading (g)	Maximise	30	50	36
E: Perforations	Any	0	5	5
Probability of PE Delamination	Maximise	0.9	0.999	0.96 ± 0.02
Probability of Al or PET Delamination	Maximise	0.9	0.999	0.95 ± 0.03

**Table 5:** Table recording the recovery of solvents and delaminated materials following experiments in the optimisation study.

#	Batch	Recovered Solvent (g)	Mass of Delaminated Materials Recovered (g)
1	60 °C	890.8	30.1
2		865.7	49.8
3		869.2	41.4
4		885.3	50.7
5		-	29.8
6	70 °C	850.0	40.5
7		867.5	50.3
8		884.2	39.7
9		-	27.4
10	80 °C	863.9	40.3
11		831.0	50.6
12		916.5	31.4
13		843.4	29.9
14		-	49.7

**Table 6:** Table of FTIR spectral absorption bands.

	Wavenumber/cm^−1^	Group	Mode	Ref.
PE	2913	C-H	stretch	[24]
	2848			
	1470	CH_2_	bending	
	718	CH_2_	rocking	
PET	1713	C=O	stretch	[25]
	1409	benzene ring	stretch	
	1340	CH_2_, *trans*	wagging	
	1241	C-C-O	asymmetric stretch	
	1092	C-O-C	stretch	
	1016	C-H, aromatic	bending	
	723	benzene ring	bending	

**Table 7:** Thermal analysis traces for PE films, (a) DSC and (b) TGA, and PET films, (c) DSC and (d) TGA, from delamination trials #5, #8, and #13.

#	Material	T_m_/°C	ΔH_m_/°C	Δχ_c_/%	T_d_/°C
5	PE	123	−138	47.5	458
8		121	−140	48.3	461
13		121	−134	46.2	462
5	PET	257	−44	31.4	416
8		256	−40	28.5	417
13		260	−45	32.1	416

## Data Availability

The original contributions presented in the study are included in the article; further inquiries can be directed to the corresponding author.
